# Seoul Virus in Pet and Feeder Rats in The Netherlands

**DOI:** 10.3390/v13030443

**Published:** 2021-03-10

**Authors:** Tryntsje Cuperus, Ankje de Vries, Tabitha E. Hoornweg, Manoj Fonville, Ryanne I. Jaarsma, Marieke Opsteegh, Miriam Maas

**Affiliations:** Centre for Infectious Disease Control, National Institute for Public Health and the Environment, Postbus 1, 3720 BA Bilthoven, The Netherlands; tryntsje.cuperus@rivm.nl (T.C.); ankje.de.vries@rivm.nl (A.d.V.); t.e.hoornweg@uu.nl (T.E.H.); manoj.fonville@rivm.nl (M.F.); ryanne.jaarsma@rivm.nl (R.I.J.); marieke.opsteegh@rivm.nl (M.O.)

**Keywords:** Seoul virus, orthohantavirus, rat

## Abstract

Seoul virus (SEOV) is a zoonotic orthohantavirus carried by rats. In humans, SEOV can cause hemorrhagic fever with renal syndrome. Recent human SEOV cases described in the USA, United Kingdom, France and the Netherlands were associated with contact with pet or feeder rats. The prevalence of SEOV in these types of rats is unknown. We collected 175 pet and feeder rats (*Rattus norvegicus*) from private owners, ratteries and commercial breeders/traders in the Netherlands. Lung tissue of the rats was tested using a SEOV real-time RT-qPCR and heart fluid was tested for the presence of antibodies against SEOV. In all three investigated groups, RT-qPCR-positive rats were found: in 1/29 rats from private owners (3.6%), 2/56 rats from ratteries (3.4%) and 11/90 rats from commercial breeders (12.2%). The seroprevalence was largely similar to the prevalence calculated from RT-qPCR-positive rats. The SEOV sequences found were highly similar to sequences previously found in domesticated rats in Europe. In conclusion, SEOV is spread throughout different populations of domesticated rats.

## 1. Introduction

Seoul virus (SEOV) is a zoonotic virus (genus *Orthohantavirus*, family *Hantaviridae*) carried by rats. Rats infected with SEOV are asymptomatic, but can transmit the virus to humans via their saliva, droppings, urine or aerosols from infected bedding. In humans, SEOV can cause mild flu-like symptoms, but also hemorrhagic fever with renal syndrome (HFRS) [1] HFRS is an acute disease characterized by fever, loin pain and varying degrees of hemorrhagic manifestations and acute kidney injury (AKI). It is estimated that about 25% of the 150,000 HFRS cases that are reported worldwide are caused by SEOV [2,3].

Over the last decade, interest in SEOV in the Western world has resurged after the description of an increasing number of human SEOV infections [4]. Many of these cases, in for example the USA, the UK, France and the Netherlands, were associated with contact with pet or feeder rats [5,6,7,8]. In all cases, the SEOV RNA found in the rats was indistinguishable from the strains detected in the patients. In the case of the first confirmed human patient in the Netherlands, 40 adult rats from the breeding farm concerned were tested and in all adult rats SEOV RNA was detected [5]. This was similar to a case from the UK, where 81% of the rats collected at a breeding farm connected to a SEOV patient were found to be SEOV RNA positive [9]. Recent prevalence studies have also been performed in wild brown rats in the UK, France and the Netherlands [10,11,12,13]. The prevalence of antibodies and/or SEOV RNA detected in these studies ranged from 0–19%.

The spread of SEOV in the general domesticated rat population is unknown. We therefore set up a study collecting pet and feeder rats in the Netherlands from three different sources: pet rats from private owners, rats from ratteries (small-scale, non-commercial breeders) and (feeder) rats from commercial breeders and traders. All rats were tested molecularly and serologically for SEOV to determine frequency of infection. Furthermore, at the start of the study, we compared the sensitivity of our molecular diagnostic method using fresh and frozen rat carcasses. In addition, phylogenetic analysis was performed on sequences from SEOV positive rats to gain insight into possible routes by which SEOV spreads throughout domesticated rat populations.

## 2. Materials and Methods

### 2.1. Study Design

Over a period of six months (May–October 2018), brown rats (*Rattus norvegicus*) were collected from three different sources: individual rat owners (contacted through veterinary practices); ratteries and shelters; and commercial rat breeders or traders. Individual rat owners and rattery or shelter owners were asked to donate rats that had died (only if frozen within 12 h after dying) or had been euthanized. Rats from breeders or traders were bought and euthanized for this study. Animals were packaged individually and stored at −20 °C until dissection. Only adult rats (>6 months of age) were collected as previous studies had indicated that SEOV prevalence is higher in older rats compared to young animals [5,14]. Owners and breeders were asked to complete a questionnaire detailing origin and handling of the rat(s), contact with other rats, breeding management and hygiene measures.

### 2.2. Dissection

Before dissection, rats were defrosted. Heart, lung and kidney samples were collected in RNAlater (ThermoFisher Scientific, Landsmeer, The Netherlands). Between rats, dissection materials were either replaced or soaked in chlorine for at least 5 min to avoid cross-contamination. The lung and kidney tissue was stored at −80 °C until analysis. Heart fluid was collected from the hearts by vortexing and centrifugation, as described previously [12], and stored at −20 °C.

### 2.3. Molecular Diagnostics

Total nucleic acid was isolated from lung and kidney tissue as described previously [5]. Total nucleic acid samples from lungs were screened by a real-time RT-PCR (RT-qPCR) using hantavirus genus specific primers targeting the nucleocapsid gene [15]. RT-qPCR was performed using Taqman Fast Virus 1-step Master Mix (Thermo Fisher Scientific, Landsmeer, The Netherlands). Samples with a Cq value <40 were considered positive. When a rat was found to be RT-qPCR-negative, but serologically positive, an additional piece of lung and kidney tissue were tested for confirmation. From a selection of the positive samples (with a maximum of two animals per positive location), S and L gene segments were amplified for sequencing using PCRs that have been described previously [5,16]. PCR fragments were purified with ExoSAP-IT PCR clean-up (Isogen Life Science, Utrecht, The Netherlands) and sequenced by Baseclear (Leiden, The Netherlands). Multiple sequence alignments for SEOV partial L segment (333nt) and S segment (272nt) sequences were obtained with MAFFT algorithm. Maximum likelihood phylogenetic trees were generated by IQtree [17] with 10,000 ultrafast bootstrap replicates [18]. Final trees were visualized in the FigTree v.1.4.4. program [19].

### 2.4. Serology

Antibodies in rat heart fluid were detected using a human SEOV ELISA (Hantavirus Dobrava/Hantaan IgG Elisa; Progen Biotechnik GmbH, Heidelberg, Germany), adapted to enable detection in rats [20]. Rabbit-α-rat horseradish peroxidase-labelled IgG (Sigma-Aldrich Chemie B.V., Zwijndrecht, The Netherlands) was used as a conjugate at a 1:5000 dilution. Two different methods were used to determine the percentage of seropositive rats. First, seropositivity of individual rats was determined as an optical density (OD) value above the cut-off value, namely the average OD of a negative control rat population [21] +/− 3 × standard deviation (SD). The seroprevalence was subsequently calculated as the proportion of seropositive rats in the sampled population. Alternatively, the seroprevalence was estimated directly from the distribution of logOD-values using the binary mixture model as described by Swart et al. [21]. Heart fluid samples from RT-qPCR-negative animals with ELISA values above the described cut-off were tested with a virus neutralization test (VNT) as described by Hoornweg et al. [22], where neutralization titers of ≥40 were considered positive.

### 2.5. Comparison of RT-qPCR Detection in Fresh and Frozen Tissue

From two breeding farms on which SEOV circulation had previously been confirmed, lung samples were collected from 10 rats shortly after euthanization and again from the same rats after >2 weeks of freezing. The samples were tested for SEOV by the RT-qPCR. Results (RT-qPCR positive or negative) of the two types of samples were compared using a Pearson’s Χ^2^-test.

### 2.6. Ethics Statement

The procedures concerning the rats from commercial breeders and traders, which had been euthanized for the purpose of the study, were approved by the Central Authority for Scientific Procedures on Animals (AVD3260020172104). The rats from private rat owners and ratteries had all died or had been euthanized already and therefore no approval was necessary. Owners, however, signed a consent form for the use of their rat(s) in this study. Data were collected according to the General Data Protection Regulation (GDPR).

## 3. Results

### 3.1. Rats

In total, 175 rats were collected and tested (Table 1). All rats were adult animals (>6 months) with an average weight of 329 g (range 170–470 g). The age of the animal was provided for most of the collected pet rats from private owners and rats from ratteries. The average age in these groups was 22 months (range 7–42 months).

### 3.2. SEOV Infections

Of the 175 rats that were tested, 14 animals were found to be SEOV positive by RT-qPCR on lung tissue. This included one pet rat from a private owner and two rats from one rattery (Table 2). In total, this rattery had submitted eight rats. From the commercial breeders and traders, two breeding farms were found to be positive. Both breeders had sent in ten animals of which, respectively, five and six rats were SEOV positive by RT-qPCR.

**Figure 1:**
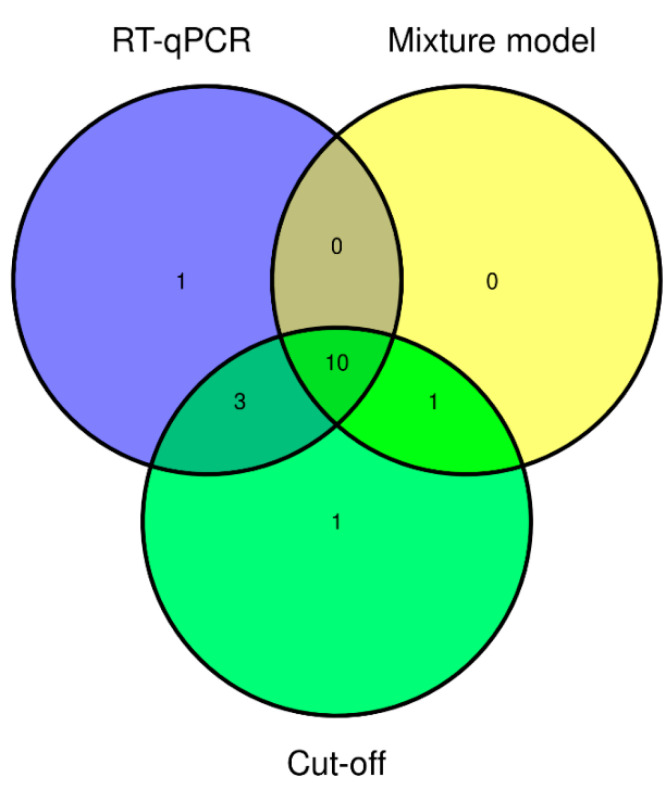
Venn diagram showing the overlap between positive results of SEOV RT-qPCR and ELISA using two different methods to determine seropositivity. Cut-off: average OD of negative control population +/− 3 × SD.

Using the cut-off of the average OD of a negative control rat population +/− 3 × SD, 15 animals were considered to be seropositive (Table 2). All 14 RT-qPCR positive rats except one were seropositive (Figure 1). In addition, two RT-qPCR negative rats, coming from a commercial breeder and a rat shelter, were seropositive. The heart fluid from these two rats was tested in the VNT for confirmation. Heart fluid from one rat showed a positive reaction in the VNT (titer 40). This rat originated from one of the positive commercial breeders, thus this finding did not result in a higher number of locations with SEOV circulation. Heart fluid from the other rat was considered VNT negative, although the first dilution (1:40) could not be read due to high levels of background signal. From these two rats, also an additional piece of lung and kidney tissue was tested by RT-qPCR. No SEOV virus was detected in these additional tissue samples.

When seropositivity was based on the binary mixture model, 11 animals had a predicted possibility to be seropositive (Pr(pos)) above 0.50 (Table 2). Four RT-qPCR positive rats had a Pr(pos) lower than 0.50 (Figure 1). In addition, one RT-qPCR negative rat had a Pr(pos) > 0.50. This was the same rat described above that showed a positive VNT reaction.

### 3.3. Questionnaires

From the questionnaires completed by the private owners of pet rats (*n* = 29), it could be concluded that the origin of these rats was very diverse. Rats had been bought from another private owner (24%), a rattery (21%), a pet store (14%) or originated from an animal shelter (24%). Over one fifth of the collected rats (21%) had regular contact with rats from other households, for example during exchanges for breeding or at pet shows. When asked about hygiene measures, most owners (90%) answered that they washed their hands after cleaning the cage. Washing of hands after touching the rats was practiced much less, only by 28% of the owners. The cage was often cleaned in a well-ventilated room (69%), but wetting the bedding before cleaning was hardly ever practiced.

The rats collected from the ratteries and shelters (*n* = 56 rats from seven locations) had mostly been bred by the rattery themselves or purchased from another rattery. Again about 20% of the rats came into contact with pet rats from outside the rattery. There was no contact between rattery rats and wild rats. All ratteries mentioned changing the bedding of the cages weekly and cleaning the cages with detergent. Similar to private owners, six out of seven rattery owners washed their hands after cleaning the cage, but only three out of seven did so after touching the rats. Wetting the bedding before cleaning the cage was not practiced by any of the participating ratteries.

The participating commercial breeding farms (*n* = 9) differed in size: from fewer than 100 to 5000 adult rats. Three out of the seven breeders mentioned buying rats from abroad, specifically Poland, Hungary and the Czech Republic. The two participating traders mentioned they mostly purchased their rats from feeder rat breeders in the Netherlands. Only two of the breeders reported contact between their own rats and rats from other farms, while three breeders mentioned that contact between their rats and wild rats was possible. All breeders mentioned that they changed the bedding of the cages every week. All breeders but one practiced washing hands after contact with the rats and all regularly washed their hands after cleaning a cage. Wearing a mouth mask while working with the rats was practiced at only two breeding farms and none of the farms practiced wetting the bedding before cleaning the cage.

### 3.4. Phylogenetics

**Figure 2:**
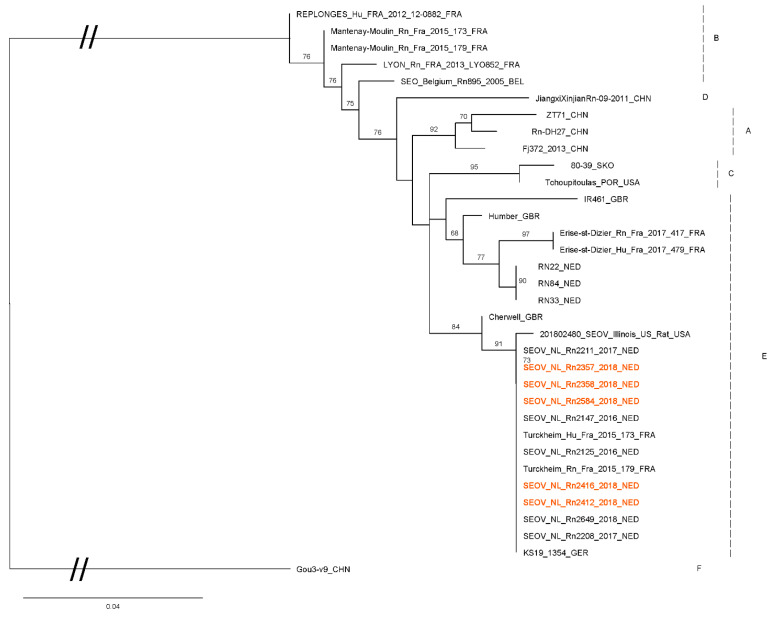
Maximum likelihood phylogenetic tree for SEOV partial S segment (274nt) sequences. Sequences from this study are shown in red (Genbank accession numbers MT993920-MT993924). The quality of the sequences from the two positive rattery rats was too low to include in this comparison. Numbers along branches are bootstrap values, only bootstrap support of >70% are shown. Scale bar indicates nucleotide substitutions per site. Letters behind branches refer to groups of SEOV sequences as described by Kim et al. [23]. At the end of the strain names the country of origin is given: BEL, Belgium; CHN, China; FRA, France; GBR, Great Britain; GER, Germany; SKO, South Korea; NED, The Netherlands; and USA, United States.

**Figure 3:**
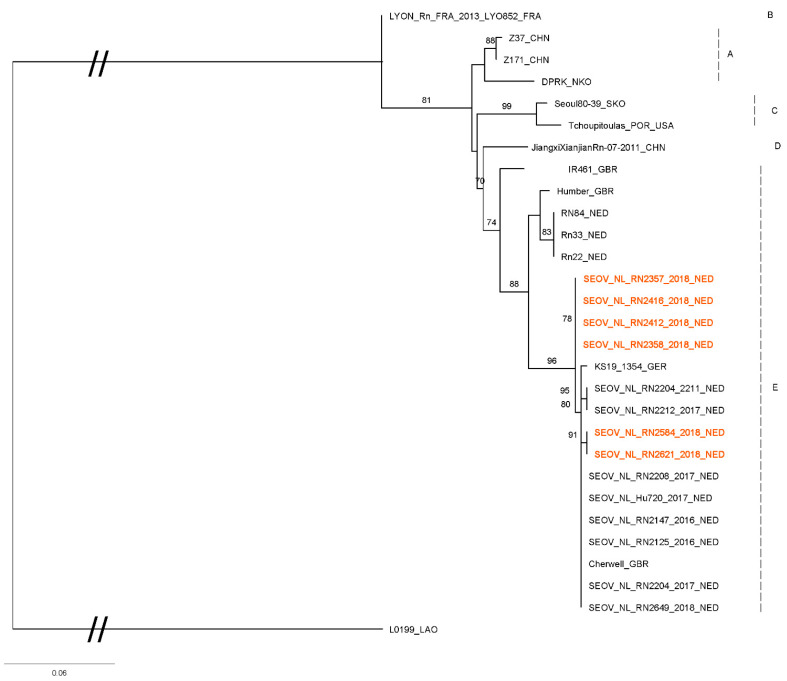
Maximum likelihood phylogenetic tree for SEOV partial L segment (333nt) sequences, 2008 Rattus tanezumi SEOV (L0199) from Laos (HQ992814) was used as an outgroup. Sequences from this study are shown in red (Genbank accession numbers MT993925-993630). The quality of one of the sequences from the rattery rats was too low to include in this comparison. Numbers along branches are bootstrap values, only bootstrap support of >70% are shown. Scale bar indicates nucleotide substitutions per site. Letters behind branches refer to groups of SEOV sequences as described by Kim et al. [23]. At the end of the strain names the country of origin is given: BEL, Belgium; CHN, China; FRA, France; GBR, Great Britain; GER, Germany; LAO, Laos; NED, the Netherlands; NKO, North Korea; SKO, South Korea; and USA, United States.

From a selection of RT-qPCR positive rats from this study (*n* = 7): two from each positive breeder and the positive rats from the rattery and private owner), the S- and L-segment of SEOV were partially sequenced (274 bp and 347 bp, respectively). Sequences were compared with SEOV sequences previously collected by our institute and sequences from Genbank (Figure 2 and Figure 3). The sequences obtained in this study all are very similar to each other and to sequences previously found in the Netherlands, UK (Cherwell) and France (Turckheim).

### 3.5. Comparison of RT-qPCR Detection in Fresh and Frozen Material

Using rats from two breeding farms, fresh lung samples were compared with lung samples that had been frozen for two weeks. In only one of the two farms, SEOV positive animals were found. Six animals had SEOV RT-qPCR positive lung tissue when tested fresh and five animals were positive when tested after freezing (Table 3). Four rats tested positive in both sets of tissues. The number of positive animals was not statistically different between the two sample types (Pearson’s Χ^2^-test, *p* = 0.65).

## 4. Discussion

To our knowledge, this is the first prevalence study about SEOV in diverse populations of domesticated rats that were not linked to human SEOV cases. In this study, we found SEOV positive animals in all three of the studied groups of rats: rats from private owners collected through veterinarians, rats from ratteries and shelters and rats from commercial breeding farms and traders. The percentages of infected rats found are difficult to interpret as high or low as no comparable studies are known. Previously published studies looking at SEOV in pet and feeder rats found very high prevalences (80–100%) [5,9]. However, in each of these two studies rats from a single breeding farm that was linked to human SEOV patients, were tested. Comparing those prevalences with the prevalences found in our study, it may be hypothesized that when human SEOV patients are linked to a colony of rats, there is (or has been) a high infection pressure, causing most of the rats to become positive. In a recent study from the USA, an association was found between increasing rat IgG or RT-qPCR prevalence and the occurrence of human SEOV infections [24]. It is noteworthy that one of the positive breeding farms in our study (where 5/10 rats were positive), had been associated with two human SEOV cases in 2017. The source investigation performed at that time showed that 10/10 adult rats collected from this farm were SEOV positive.

Official data about the number of rat owners and breeders in the Netherlands are not available. From our experience, it appears that a substantial number of rat breeders are not registered, including large breeders with over 1000 parent rats. Because an overview of the numbers of pet rats and feeder rats is missing, the percentages of infected rats found in this study may not be representative of the true national prevalence. The numbers we present here should thus be seen as an indication of the prevalence and distribution of SEOV in domesticated rats in the Netherlands. One group of domesticated rats, namely the laboratory rats, was not investigated in this study, although human SEOV cases have been described among personnel working with infected laboratory rats [25,26,27]. However, since laboratory rats are nowadays checked annually for orthohantavirus according to the guidelines of the Federation of European Laboratory Animal Science Associations [28], we decided not to include this group.

The obtained sequences are very similar to previously published SEOV sequences from wild and domesticated rats in different European countries and they cluster together in what is called “group E” or “lineage 9” [10,23,29]. Within “group E”, sequences found in this study cluster with sequences found previously in pet rats and feeder rats from the Netherlands, the UK and France [7,10]. Sequences found in wild rats in Europe are found in a different sub-cluster in group E.

Import of rats from abroad was mentioned in questionnaires from all sources of rats. From the five participating ratteries two mentioned contact with rats from abroad or having bought rats from other countries. One of those was the rattery that tested SEOV positive and one of the positive rats was bought from abroad (Germany). Additionally, two breeding farms mentioned importing rats from abroad, including one of the positive commercial breeding farms (import from Czech Republic). From private owners, only one rat came from abroad (Belgium), which happened to be the one positive rat from this source. Though noteworthy, the relatively small number of positive rats did not allow for a quantification of the influence of import from abroad, and this should be further investigated in future studies. Because of the minimal genetic differences between SEOV strains within Europe, it was not possible to trace the spread of SEOV to and within the Netherlands.

In this study, molecular diagnostics on fresh and frozen lung tissues from rats were compared. Though with fresh tissue 6/10 rats were found RT-qPCR-positive, compared to 5/10 with frozen tissue, the difference was not statistically significant. The difference, if not due to coincidence, may be caused by the freezing, or due to a heterogeneous distribution of SEOV over the organ. This should be further studied. Being able to use frozen rats for SEOV diagnostic can make future research and source finding (in the case of human patients) logistically much easier. However, it may be sensible to test two different parts of an organ in future studies to account for potential heterogeneous distribution of SEOV.

In this study, we used two methods to determine seropositivity; using a cut-off based on a negative control rat population and by binary mixture modelling. The serologic results based on the cut-off method showed the highest agreement with the results from the RT-qPCR. However, using a binary mixture model is theoretically more appropriate and has multiple advantages such as measures of uncertainty and the proper treatment of censored values (e.g., negative OD values) as discussed more extensively in the article of Swart et al. [21]. With both methods, most seropositive rats were also RT-qPCR positive. This observation is in accordance with the fact that hantaviruses generally cause persistent infections in rodent hosts with antibodies and virus present simultaneously [14,30]. Clearance of SEOV in rats is also possible, especially when animals are infected at a later age [30]. In our study we observed one RT-qPCR-negative rat where seropositivity was confirmed by VNT, underlining the added value of the VNT for rats that have disagreeing results.

In conclusion, we found SEOV in three different groups of domesticated rats in the Netherlands: rats from private owners, ratteries and commercial breeding farms and traders. Control of SEOV in the Netherlands would be complicated because many rat breeders and owners are not registered, and it is not yet known what measures could be effective to reduce transmission of the virus among rats. To prevent new human SEOV cases, we therefore suggest improving public education on SEOV [31]. From our questionnaires, it became clear that improvements in hygiene measures are possible; especially washing hands after contact with the rats and wetting of the bedding was practiced by only a limited number of pet rat and rattery owners. Improvement of hygiene measures should therefore be the main focus of communication and education to (prospective) rat owners.

## Figures and Tables

**Table 1:** Sources of rats collected for this study.

Source	Number of Participants	Total Number of Rats Collected	Number of Rats per Participant (Range)
Private owners via veterinarians	8 veterinarians ^1^	29	2–11
Ratteries and rat shelters	5 ratteries, 2 shelters	56	5–10
Commercial breeders and traders	9	90	10

^1^ The number of individual rat owners is unknown as it was not compulsory for them to provide contact details.

**Table 2:** Results of Seoul virus (SEOV) diagnostics.

Source	Total Number of Rats	Nr. of Rats RT-qPCR+ (Percentage)	Nr. of Rats ELISA+ Based on Cut-Off ^1^ (Percentage)	Nr. of Rats ELISA+ Based on Mixture Model (Percentage)
Private owners via veterinarians	29	1 (3.4%)	1 (3.4%)	1 (3.4%)
Ratteries and rat shelters	56	2 (3.6%)	3 (5.4%)	1 (1.8%)
Commercial breeders and traders	90	11 (12.2%)	11 (12.2%)	9 (10.0%)

^1^ Cut-off: average OD of negative control population +/− 3 × SD.

**Table 3:** Comparison of SEOV RT-qPCR results on fresh versus frozen lung tissue from ten rats.

Rat ID	RT-qPCR Result on Fresh Tissue (Cq Value)	RT-qPCR Result on Frozen Tissue (Cq Value)
RN2352	-	-
RN2353	+(27.83)	-
RN2354	+(30.11)	+(26.53)
RN2355	-	-
RN2356	-	+(28.38)
RN2357	+(25.61)	+(25.72)
RN2358	+(23.68)	+(22.61)
RN2359	+(26.67)	+(27.91)
RN2360	-	-
RN2361	+(29.84)	-

## Data Availability

Data is contained within the article.
